# Healthcare costs associated with comorbid cardiovascular and renal conditions among persons with diabetes, 2008–2019

**DOI:** 10.1186/s13098-022-00957-z

**Published:** 2022-11-28

**Authors:** Chintal H. Shah, Chintan V. Dave

**Affiliations:** 1 Department of Pharmaceutical Health Services Research, University of Maryland School of Pharmacy, 220 N. Arch Street, 12th floor, Baltimore, MD 21201 USA; 2 Center for Pharmacoepidemiology and Treatment Science, Institute for Health, Health Care Policy and Aging Research, Rutgers University, New Brunswick, NJ USA; 3 Department of Pharmacy Practice and Administration, Ernest Mario School of Pharmacy, Rutgers University, Piscataway, NJ USA

**Keywords:** Diabetes, Heart failure, Kidney disease, Atherosclerotic cardiovascular disease, ASCVD, Costs, Cardiorenal, MEPS

## Abstract

**Background:**

There is paucity of data examining healthcare costs among persons with comorbid diabetes and cardiorenal conditions.

**Objective:**

To elucidate the longitudinal trends and quantify the incremental healthcare costs associated with the following cardiorenal conditions: atherosclerotic cardiovascular disease (ASCVD), heart failure (HF), and kidney disease, among persons with diabetes.

**Methods:**

Medical Expenditure Panel Survey data (2008–2019) were used to identify adults with diabetes and comorbid cardiorenal conditions. Overall, medical and pharmaceutical costs were ascertained (in 2019 US dollars). Analyses were adjusted for 14 variables using a two-part regression model.

**Results:**

Among 32,519 adults with diabetes, the mean (standard error [SE]) annual healthcare costs were $13,829 ($213), with medical and prescription components contributing $9301 ($172) and $4528 ($98), respectively. Overall healthcare costs rose by 26.8% from $12,791 (2008–2009) to $16,215 (2018–2019) over the study period, driven by 42.5% and 20.3% increase in pharmaceutical and medical spending, respectively. Similar trends were observed for subgroup of persons with cardiorenal conditions. Compared to their counterparts without cardiorenal conditions and prior to adjustment, persons with ASCVD, HF and kidney disease incurred healthcare costs that were approximately 2.2, 3.3, and 2.7 times greater. After adjustment, comorbid ASCVD, HF and kidney disease were associated with annual excess spending of $8651 (95% CI $7729–$9573), $9373 (95% CI $9010–$9736), and $9995 (95% CI $8781–$11,209), respectively.

**Conclusions:**

Study results are generalizable to non-institutionalized US persons. Healthcare costs associated with the management of diabetes are high—especially among those with comorbid cardiorenal conditions, and have risen in recent years.

**Supplementary Information:**

The online version contains supplementary material available at 10.1186/s13098-022-00957-z.

Diabetes, a highly prevalent condition affecting 11.3% of the US population [1], is a key driver of healthcare resource utilization, premature mortality, lost productivity, and reduced quality of life [2–4]. Consequently, it poses a significant economic burden on the US healthcare system. The direct and overall annual costs attributable to diabetes in 2017 alone were estimated to exceed US $230 and $320 billion respectively, representing a 26% increase from 2012 [5].

Due to the shared pathophysiological mechanisms underpinning the development of diabetes and cardiorenal conditions as well as the micro- and macro-vascular consequences of unmitigated chronic hyperglycemia, comorbid conditions such as atherosclerotic cardiovascular diseases (ASCVD), heart failure (HF), and chronic kidney disease (CKD) affect more than one-in-three persons with diabetes [6, 7]. Compared to diabetes alone, the co-occurrence of diabetes and cardiorenal diseases augurs a clinical course characterized by greater insulin resistance [8], accelerated disease progression [10], escalation in clinical complexity, and an increased risk of cardiovascular events, end-stage renal disease and all-cause mortality [11, 12].

The costs associated with the management of comorbidities among persons with diabetes are substantial, and are estimated to comprise of > 90% of all healthcare spending [5]. Despite the high prevalence of cardiorenal conditions among persons with diabetes, remarkably little is known regarding the incremental healthcare costs imposed by such conditions. Accordingly, using a nationally representative database from 2008 to 2019, the objectives of our study were: (i) to elucidate the longitudinal trends in healthcare spending among persons with (a) diabetes overall, (b) diabetes without cardiorenal conditions, (c) diabetes with ASCVD, (d) diabetes with HF, and (e) diabetes with kidney disease; and (ii) to quantify the unadjusted and adjusted incremental costs associated with these cardiorenal conditions on the overall, medical, and prescription drug spending.

## Methods

### Data sources and study population

Data from the 2008–2019 Medical Expenditure Panel Survey (MEPS)—provided by the Agency for Healthcare Research and Quality—were utilized in this study. The MEPS database is a publicly available, nationally representative sample containing deidentified data on individuals from the subset of households that responded to the National Health Interview Survey conducted by the National Center for Health Statistics [13]. A new panel of approximately > 39,000 community residing individuals are surveyed every year and information on demographic and socioeconomic characteristics, healthcare utilization, health status and functioning, along with other healthcare related information, is collected.

The study sample was comprised of all adults ≥ 18 years between 2008 and 2019 with a diagnosis of diabetes. Data from both the full-year consolidated data files and the medical conditions files were used to ascertain the presence of medical conditions, thereby maximizing the sensitivity of our definitions. The presence of diabetes was determined based on an affirmative self-reported answer to the question of whether a person had ever been diagnosed with diabetes (excluding gestational diabetes) or the presence of international classification of disease (ICD)-9 or ICD-10 codes corresponding to diabetes (see Additional file 1: Appendix Table A1 for all definitions). In MEPS data, the first three digits of the ICD codes are available as ICD-9-CM versions till 2015, and as ICD-10-CM versions from 2016 onwards.

**Fig. 1:**
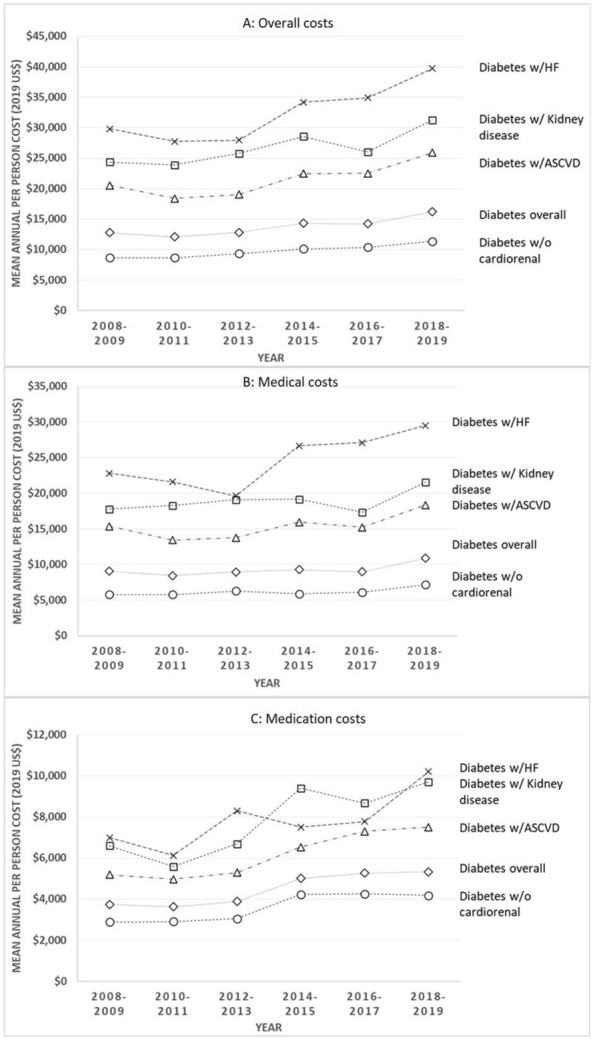
Unadjusted trends in the overall (**A**), medical (**B**), and medication (**C**) spending in persons with diabetes and cardiorenal comorbidities. Analyses were not adjusted for covariates. Survey weights and procedures were utilized. See text for details on ascertainment of spending and definitions of cardiorenal conditions. ASCVD, Atherosclerotic cardiovascular disease; HF, Heart failure

### Patient characteristics and cardiorenal conditions

Diagnosis of ASCVD required individuals to have evidence of coronary heart disease, myocardial infarction or angina, stroke, or peripheral vascular disease (either through self-report or ICD-codes) [14]. As the survey did not collect patient information on HF status, presence of HF was determined using ICD-codes only. The presence of kidney disease was determined through self-reports using previously described methods [15], as the first three digits of the ICD codes alone are insufficient to stratify individuals according to their kidney function.

We examined and reported pertinent patient characteristics among individuals with (a) diabetes overall, (b) diabetes without cardiorenal comorbidities, (c) diabetes with ASCVD, (d) diabetes with HF, and (e) diabetes with kidney disease. These characteristics included age, sex, race and ethnicity, marital status, familial income, geographical region, insurance coverage, and other comorbid conditions. We also reported the unadjusted healthcare-, medical-, and prescription-costs across these five subgroups.

### Healthcare costs

The primary outcome of interest was the annual overall healthcare related costs; these overall costs were also further stratified into two mutually-exclusive contributing components corresponding to prescription drug costs and overall healthcare costs besides prescription drug costs (“medical costs”). Costs were adjusted to 2019 values using the Personal Health Care Expenditure (PHCE) component of the National Health Expenditure Accounts, provided by the U.S. Department of Health and Human Services [16].

### Statistical analysis

The period from 2008 to 2019 was segmented into biennial calendar intervals, and the mean healthcare, medical and prescription costs were reported for the five subgroups of interest for each two-year period.

To quantify the additive costs imposed by cardiorenal conditions, the incremental expenditures regression-based approach was employed [17]. The dependent variables of interest were the all-cause overall healthcare-, medical-, and prescription- costs, and the primary independent variables of interest were the presence of ASCVD, HF, and kidney disease. To avoid overadjustment (i.e., adjusting away the true effect of the cardiorenal conditions on healthcare spending), we first estimated the age, sex and calendar year only adjusted regression models. Thereafter, we estimated fully-adjusted models controlling for the effects of age, insurance coverage, sex, race/ethnicity, marital status, family income, region, year, and select non-cardiorenal comorbidities. For all regression-based analyses, the two-part model with delta estimation was used [18, 19]. Briefly, the first part of the regression model was a logistic regression where the outcome of interest was the presence or absence of costs. The second part was a generalized linear model (GLM), with a log link function, and either a Poisson, gamma, or inverse gaussian distribution as determined by the modified Park test [20–22].

The appropriate survey weights and design methodologies were utilized to account for the complex survey nature of the data and ensure the national representativeness of the study estimates [23]. All analyses were conducted using SAS 9.4 and StataMP 16. The level of confidence and statistical significance was determined at the 95% level.

## Results

### Patient characteristics

Between 2008 to 2019, we identified 32,519 eligible persons with diabetes (Table 1), out of which 9599 (29.5%), 952 (2.9%), and 2820 (8.7%) had comorbid ASCVD, HF and kidney disease, respectively.

**Table 1:** Patient characteristics by diabetes, ASCVD, HF and kidney disease status

	Diabetes overall (n = 32,519)	Diabetes w/o cardiorenal^a^ (n = 21,290)	Diabetes w/ASCVD (n = 9599)	Diabetes w/HF (n = 952)	Diabetes w/kidney disease (n = 2820)
*Sociodemographic and other characteristics, n (%)*					
Male	14,923 (45.9)	9261 (43.5)	4937 (51.4)	460 (48.3)	1300 (46.1)
Age, mean (SD)	60.5 (14.0)	57.8 (14.0)	66.5 (12.0)	67.1 (12.1)	63 (13.4)
Age 18–44	4415 (13.5)	3745 (17.6)	419 (4.4)	37 (3.9)	284 (10.1)
Age 45–65	14,781 (45.5)	10,441 (49.0)	3594 (37.4)	356 (37.4)	1181 (41.8)
Age > 65	13,323 (41.0)	7104 (33.4)	5586 (58.2)	559 (58.7)	1355 (48.1)
Race/ethnicity: Non-Hispanic Black	5056 (23.8)	7794 (24.0)	2288 (23.9)	294 (30.9)	738 (26.2)
Race/ethnicity: Hispanic	5926 (27.8)	8295 (25.5)	1971 (20.5)	101 (10.6)	640 (22.7)
Race/ethnicity: other	10,308 (48.4)	16,430 (50.5)	5340 (55.6)	557 (58.5)	1442 (51.1)
Married	17,122 (52.7)	11,667 (54.8)	4701 (49.0)	396 (41.6)	1289 (45.7)
Year, 2008–2011	9878 (30.4)	6382 (30.0)	2976 (31.0)	310 (32.6)	883 (31.3)
Year, 2012–2015	11,320 (34.8)	7391 (34.7)	3346 (34.9)	327 (34.4)	1040 (36.9)
Year, 2016–2019	11,321 (34.8)	7517 (35.3)	3277 (34.1)	315 (33.0)	897 (31.8)
Family income: poor	6960 (21.4)	4136 (19.4)	2413 (25.1)	240 (25.2)	773 (27.4)
Family income: near poor	2349 (7.2)	1394 (6.5)	825 (8.6)	77 (8.1)	241 (8.6)
Family income: low income	5777 (17.8)	3658 (17.2)	1785 (18.6)	190 (20.0)	574 (20.4)
Family income: middle income	9379 (28.8)	6312 (29.7)	2625 (27.4)	262 (27.5)	753 (26.6)
Family income: high income	8054 (24.8)	5790 (27.2)	1951 (20.3)	183 (19.2)	479 (17.0)
Region: Northeast	5054 (15.5)	3242 (15.2)	1641 (17.1)	109 (11.5)	327 (11.6)
Region: West	7579 (23.3)	5372 (25.2)	1797 (18.7)	157 (16.5)	466 (16.5)
Region: Midwest	6035 (18.6)	3847 (18.1)	1910 (19.9)	249 (26.2)	1361 (48.3)
Region: South	13,851 (42.6)	8829 (41.5)	4251 (44.3)	437 (45.8)	666 (23.6)
Insurance coverage: Public only	13,623 (41.9)	7589 (35.7)	5201 (54.2)	548 (57.6)	1574 (55.8)
insurance coverage: any private	15,982 (49.1)	11,397 (53.5)	3927 (40.9)	368 (38.6)	1047 (37.1)
Insurance coverage: uninsured	2914 (9.0)	2304 (10.8)	471 (4.9)	36 (3.8)	199 (7.1)
*Comorbid conditions, n (%)*					
ASCVD	9599 (29.5)	NA	NA	718 (75.4)	1371 (48.6)
Myocardial infarction	3936 (12.1)	NA	3936 (41.0)	369 (38.8)	606 (21.5)
Stroke	3819 (11.7)	NA	3819 (39.8)	276 (29.0)	617 (21.9)
Angina	2451 (7.5)	NA	2451 (25.5)	223 (23.4)	410 (14.5)
Heart failure	952 (2.9)	NA	718 (7.5)	NA	227 (8.1)
Kidney disease	2820 (8.7)	NA	1371 (14.3)	227 (23.8)	NA
Arthritis	16,358 (50.3)	9185 (43.1)	6234 (64.9)	667 (70.1)	1848 (65.5)
Chronic obstructive pulmonary disease	3156 (9.7)	1313 (6.2)	1641 (17.1)	272 (28.6)	468 (16.6)
Asthma	4662 (14.3)	2619 (12.3)	1791 (18.7)	222 (23.3)	517 (18.3)
high cholesterol	23,071 (71.0)	13,831 (65.0)	7981 (83.1)	779 (81.8)	2311 (82.0)
Cancer	5132 (15.8)	2688 (12.6)	2158 (22.5)	235 (24.7)	600 (21.3)
Depression	4827 (14.8)	2686 (12.6)	1839 (19.2)	253 (26.6)	557 (19.8)
*Healthcare spending, mean (SE)*^b^					
Overall costs	13,829 (213)	9844 (196)	21,542 (501)	32,654 (1571)	26,652 (1060)
Medical costs	9301 (172)	6203 (153)	15,372 (400)	24,837 (1,465)	18,844 (779)
Prescription costs	4528 (98)	3642 (113)	6170 (180)	7817 (476)	7808 (495)

ASCVD, atherosclerotic cardiovascular disease; HF, heart failure

^a^Comorbidities of interest were ASCVD, HF, and kidney disease

^b^Analyses were not adjusted for covariates. Survey weights and procedures were utilized. See text for details on ascertainment of spending and definitions of cardiorenal conditions

Overall, the cohort was majority female (54.1%), with a mean (standard deviation) age of 60.5 (14.0) years. Compared to their counterparts without cardiorenal conditions, those with ASCVD were older, and were more likely to be diagnosed with other chronic conditions such as arthritis, chronic obstructive pulmonary disease, asthma, high cholesterol, cancer and depression. Myocardial infarction (41.0%) and stroke (39.8%) were the most prevalent ASCVD conditions in this subgroup. Similarly, compared to those without co-occurring cardiorenal conditions, individuals with HF and kidney disease were older, and had a greater burden of comorbid conditions.

### Unadjusted healthcare costs

The mean (standard error) unadjusted, overall healthcare costs (in 2019 US dollars) for persons with diabetes were $13,829 ($213), with medical and prescription drug components contributing $9301 ($172) and $4528 ($98), respectively (Table 1). Individuals with ASCVD, HF, and kidney disease incurred healthcare expenses that were approximately 2.2, 3.3, and 2.7 times greater than those without cardiorenal diseases. Similarly, medical expenditures were 2.5, 4, and 3 times higher, while prescription drug costs were 1.7, 2.1, and 2.1 times higher, for persons with ASCVD, HF, and kidney disease, respectively. Prescription drug costs accounted for 37.0% of the total healthcare spending among persons with diabetes without cardiorenal conditions compared to 28.6%, 23.9%, and 29.3% among persons with diabetes and comorbid ASCVD, HF, and kidney disease, respectively.

The mean (95% confidence interval) overall healthcare spending for persons with diabetes increased by 26.8% from $12,791 ($12,047–$13,536) in 2008–09 to $16,215 ($15,274–$17,156) in 2018–2019 (Fig. 1; Additional file 1: Appendix Tables A2–A4), and by 31.3% from $8650 ($7974–$9327) in 2008–2009 to $11,361 ($10,642–$12,080) in 2018–2019 among persons without cardiorenal conditions. The corresponding increase in healthcare spending for patient with cardiorenal conditions—which was already higher than those without such conditions—increased further by 26.0% from $20,539 ($18,688–$22,389) to $25,878 ($23,496–$28,260), 33.3% from $29,797 ($22,834–$36,761) to $39,715 ($30,693–$48,738), and 28.4% from $24,358 ($20,494–$28,223) to $31,272 ($26,652–$35,892) among persons with ASCVD, HF and renal disease, respectively. This rise was driven by an increase in prescription drug spending which increased by greater than 40% for all groups, ranging between 42.5 and 47.3%. By contrast, over the study period, medical expenditures—while comprising of a larger component of overall healthcare costs—increased more modestly for most subgroups ranging from 19.7 to 24.4%, with the exception of HF persons for whom such spending increased by 29.4%.

### Adjusted healthcare costs

After adjusting for the effects of age, biological sex, and calendar year, the mean (95% CI) excess healthcare spending associated with ASCVD, HF, and kidney disease among persons with diabetes was $10,330 ($9376–$11,285), $11,318 ($11,089–$11,547), and $11,625 ($10,441–$12,808), respectively (Table 2). After further adjustments for demographic, socioeconomic, and clinical factors, these estimates were modestly attenuated but remained significant; diagnosis of ASCVD, HF, and kidney disease were associated with $8651 ($7729–$9573), $9373 (95% CI $9010–$9736), and $9995 ($8781–$11,209) excess per-person costs, respectively, with medical spending remaining the biggest contributing component of excess healthcare spending.

**Table 2:** Incremental costs associated with cardiorenal comorbidities among persons with diabetes^a^

	Diabetes w/o cardiorenal conditions^b^	Diabetes w/ASCVD	Diabetes w/Heart failure	Diabetes w/kidney disease
*Age, sex and calendar year adjusted models*				
Overall costs	Ref	10,330 (9376–11,285)^c^	11,318 (11,089–11,547)^c^	11,625 (10,441–12,808)^d^
Medical costs	Ref	7833 (7096–8571)^c^	9734 (8703–10,766)^c^	8413 (7555–9271)^d^
Prescription costs	Ref	2454 (2140–2769)^d^	3565 (3056–4074)^e^	3281 (2831–3731)^e^
*Fully adjusted models*^*a*^				
Overall costs	Ref	8651 (7729–9573)^c^	9373 (9010–9736)^c^	9995 (8781–11,209)^c^
Medical costs	Ref	6867 (6123–7612)^c^	8366 (7340–9392)^c^	7408 (6484–8333)^c^
Prescription costs	Ref	1769 (1501–2037)^d^	2561 (2124–2999)^d^	2676 (2295–3058)^d^

ASCVD, Atherosclerotic cardiovascular disease; HF, Heart failure

^a^Values in 2019 US dollars. All differences were significant at 95% confidence interval. Covariates in fully adjusted models include: age (10-year intervals), insurance coverage, sex, race/ethnicity, marital status, family income, region, year, and comorbid conditions (arthritis, asthma, cancer, high cholesterol, COPD, and depression)

^b^Comorbidities of interest were ASCVD, HF, and kidney disease

^c^For two-part regression: first part: logit; second part: generalized linear model (family = Poisson, link = log)

^d^For two-part regression: first part: logit; second part: generalized linear model (family = gamma, link = log)

^e^For two-part regression: first part: logit; second part: generalized linear model (family = inverse gaussian, link = log)

## Discussion

Amid the backdrop of worsening epidemiological trends in the incidence and prevalence of diabetes and co-existing cardiorenal conditions in the US, we leveraged a large, nationally representative database from 2008 to 2019 to elucidate the longitudinal trends in healthcare, medical, and pharmaceutical spending among persons with diabetes and comorbid cardiorenal conditions, and sought to quantify the incremental healthcare costs incurred by such conditions among persons with diabetes. Our study found that between 2008 and 2019, healthcare costs for persons with diabetes increased by 26.8%, driven by a 20.3% and 42.5% corresponding increase in medical and pharmaceutical spending, respectively. Compared to their counterparts without cardiorenal conditions, crude healthcare costs for persons with such comorbid conditions were found to be higher, and increased further by 26.0–33.3% over the study period. After adjustment, the incremental healthcare costs associated with the management of cardiorenal conditions ranged between $8651 and $9995, and were greatest for persons with kidney disease, followed by HF and ASCVD.

Prior data examining the costs associated with comorbid cardiorenal conditions in persons with diabetes are older, sparse, less generalizable, and descriptive when compared to our incremental regression approach. For instance, a prior study using Translating Research Into Action for Diabetes data from 1999 to 2002 reported that the mean unadjusted healthcare costs for persons with diabetes with HF were $10,630 [24]. By contrast, our study—which utilized more recent and generalizable data and adjusted for key sociodemographic and clinical factors—found that the mean unadjusted and incremental costs for such persons were $32,654 and $11,318, respectively. Another study using 2011 MEPS data reported the mean incremental healthcare costs associated with co-occurring kidney disease among persons with diabetes to be $8473, while we saw an 18% increase over these prior estimates [15]. Another study using a US administrative claims database from 2015 estimated the unadjusted healthcare costs of persons dually diagnosed with diabetes and ASCVD at $22,977 [25]. Our unadjusted healthcare costs for ASCVD were similar, though our study also included publicly insured and uninsured individuals.

We found the average overall costs for persons with diabetes were $13,829, rising from $12,791 to $16,215 over the study period. These findings coincide with a prior American Diabetes Association sponsored study which used data from 2017 and estimated the direct healthcare costs for persons with diabetes at $16,752 [5]. A major factor contributing to this rise in spending was prescription drug prices which rose by 42.5% over the study period, which may reflect the approval of several new diabetes medications during this period; the majority of them were branded and more expensive.

There exists a high burden of cardiorenal conditions among individuals with diabetes in the US, and Rowley et al. have projected that between 2015 and 2030, there is estimated be a 54% increase in the prevalence of diabetes, and a corresponding 53% rise in the total annual diabetes-related costs [26]. This study quantifies and corroborates that the costs for the management of diabetes are high and continue to rise. The potential reasons for this increased economic burden are likely multifactorial and include: an increase in the incidence and prevalence of diabetes in the US; rise in the per-person healthcare costs associated with the management of diabetes; worsening trends in the incidence and prevalence of cardiorenal multi-morbidities among persons with diabetes; and rising prescription drug costs.

The study findings have pertinent implications. To our knowledge, this investigation represents the first comprehensive effort to leverage a large, highly representative database—which included uninsured as well under- and fully-insured individuals with public or private insurance—to elucidate the longitudinal trends in healthcare, medical, and pharmaceutical costs among persons with diabetes and cardiorenal conditions. Further, the study also employed a regression based-approach to estimate the incremental costs incurred by persons with diabetes with prevalent cardiorenal conditions. The 12-year study period also enabled us to account for the changing landscape in the management of diabetes and its comorbidities; some of these changes include the introduction of new therapeutic modalities including entirely new medication classes and changes in guidelines for the management of diabetes. Study limitations are noted. First, despite measures taken by MEPS to ensure complete and accurate information, it is possible that any data collected through such means can be susceptible to recall bias [27, 28]. Second, as the sample surveyed is restricted to non-institutionalized, community residing individuals, the results are not generalizable to institutionalized individuals. Finally, prior studies have shown diabetes imposes substantial burden on direct as well as indirect costs; our study is only able to quantify the role of direct costs.

In conclusion, our investigation found that the healthcare costs associated with the management of persons with diabetes—especially those with comorbid cardiorenal conditions—are high, and continue to rise. As the landscape of diabetes management shifts from a glucocentric approach that prioritizes reductions in hemoglobin A1C to one that considers long-term cardiorenal health, continued research examining the burden and impact of such conditions will become increasingly important.

## Supplementary Information

**Additional file 1. Table A1**. Definitions for various conditions utilized. **Table A2.** Unadjusted trends in the mean (95% confidence interval) overall spending in persons with diabetes and cardiorenal comorbidities^a^. **Table A3.** Unadjusted trends in the mean (95% confidence interval) medical spending in persons with diabetes and cardiorenal comorbidities^a^. **Table A4.** Unadjusted trends in the mean (95% confidence interval) medication spending in persons with diabetes and cardiorenal comorbidities^a^

## Data Availability

Data used was publicly available data provided by the Agency for Healthcare Research and Quality and can be accessed at https://meps.ahrq.gov/mepsweb/

## Data Availability Statement

The datasets used and/or analyzed during the current study are available from the corresponding author on reasonable request.
