# Supplementary material for: Healthcare costs associated with comorbid cardiovascular and renal conditions among persons with diabetes, 2008–2019
Source: Diabetol Metab Syndr. 2022 Nov 28;14:179. doi: 10.1186/s13098-022-00957-z (PMC9703659; doi:10.1186/s13098-022-00957-z)
Supplement: Supplementary file 1 — Additional file 1. Table A1. Definitions for various conditions utilized. Table A2. Unadjusted trends in the mean (95% confidence interval) overall spending in persons with diabetes and cardiorenal comorbiditiesa. Table A3. Unadjusted trends in the mean (95% confidence interval) medical spending in persons with diabetes and cardiorenal comorbiditiesa. Table A4. Unadjusted trends in the mean (95% confidence interval) medication spending in persons with diabetes and cardiorenal comorbiditiesa [file 13098_2022_957_MOESM1_ESM.docx]

Supplemental material

Appendix A

| **Table A1: Definitions for various conditions utilized** | |  |
| --- | --- | --- |
| **Condition** | **Definition** | |
| Diabetes | Self-reported ever diabetes (excluding gestational diabetes) **OR** presence of ICD codes (ICD-9-CM: “250”/ ICD-10-CM: "E08","E09","E10","E11","E13"). |  |
| ASCVD | **Presence of any:**   - Coronary artery disease (self-reported ever coronary heart disease **OR** heart attack **OR** angina, **OR** presence of ICD codes (ICD-9-CM: “410”, “413”, “414”/ICD-10-CM: “I20”, “I21”, “I25”)) **OR** - Stroke (self-reported ever stroke **OR** presence of ICD codes (ICD-9-CM: “433”, ”434”, ”435”, ”436”, ”437”/ICD-10: “I63”, “G45”) **OR** - Peripheral vascular disease (presence of ICD codes (ICD-9: “440”, “443”/ICD-10-CM: “I70”, “I73”, “I79”)) |  |
| HF | Presence of ICD codes (ICD-9-CM: “428”/ICD-10-CM: “I50”) |  |
| Kidney disease | Self-reported kidney problems due to diabetes |  |
| Abbreviations: ASCVD: Atherosclerotic cardiovascular disease; HF: Heart failure; ICD-9-CM/ICD-10-CM: International classification of disease 9/10 clinical modification code | |  |

**Table A2:** Unadjusted trends in the mean (95% confidence interval) overall spending in persons with diabetes and cardiorenal comorbidities. ^a^

| **Year** | **Diabetes overall** | **Diabetes w/o cardiorenal** | **Diabetes w /ASCVD** | **Diabetes w/ HF** | **Diabetes w/ kidney disease** |
| --- | --- | --- | --- | --- | --- |
| **2008-2009** | $12,791  (12,047-13,536) | $8,650  (7,974-9,327) | $20,539  (18,688-22,389) | $29,797  (22,834-36,761) | $24,358  (20,494-28,223) |
| **2010-2011** | $12,064  (11,328-12,800) | $8,675  (7,967-9,382) | $18,405  (16,645-20,166) | $27,741  (22,008-33,474) | $23,886  (20,106-27,665) |
| **2012-2013** | $12,818  (11,855-13,781) | $9,344  (8,365-10,322) | $19,051  (17,061-21,042) | $27,934  (22,533-33,335) | $25,770  (20,786-30,755) |
| **2014-2015** | $14,303  (13,111-15,494) | $10,101  (8,993-11,209) | $22,478  (19,802-25,154) | $34,178  (28,070-40,286) | $28,568  (20,000-37,135) |
| **2016-2017** | $14,233  (13,333-15,134) | $10,376  (9,648-11,104) | $22,512  (20,100-24,925) | $34,883  (24,543-45,224) | $26,016  (22,909-29,123) |
| **2018-2019** | $16,215  (15,274-17,156) | $11,361  (10,642-12,080) | $25,878  (23,496-28,260) | $39,715  (30,693-48,738) | $31,272  (26,652-35,892) |

Abbreviations: ASCVD: Atherosclerotic cardiovascular disease; HF: Heart failure

^a^ Analyses were not adjusted for covariates. Survey weights and procedures were utilized. See text for details on ascertainment of spending and definitions of cardiorenal conditions.

**Table A3:** Unadjusted trends in the mean (95% confidence interval) medical spending in persons with diabetes and cardiorenal comorbidities. ^a^

| **Year** | **Diabetes overall** | **Diabetes w/o cardiorenal** | **Diabetes w /ASCVD** | **Diabetes w/ HF** | **Diabetes w/ kidney disease** |
| --- | --- | --- | --- | --- | --- |
| **2008-2009** | $9,054  (8,391-9,716) | $5,772  (5,181-6,362) | $15,355  (13,694-17,016) | $22,802  (16,331-29,274) | $17,766  (14,010-21,522) |
| **2010-2011** | $8,439  (7,744-9,134) | $5,770  (5,120-6,419) | $13,433  (11,805-15,061) | $21,623  (16,106-27,139) | $18,298  (14,785-21,810) |
| **2012-2013** | $8,938  (8,039-9,837) | $6,291  (5,393-7,189) | $13,753  (11,929-15,578) | $19,631  (14,769-24,492) | $19,080  (14,498-23,663) |
| **2014-2015** | $9,294  (8,569-10,019) | $5,876  (5,309-6,443) | $15,942  (14,215-17,670) | $26,676  (21,013-32,339) | $19,166  (14,797-23,535) |
| **2016-2017** | $8,973  (8,213-9,733) | $6,124  (5,528-6,720) | $15,207  (13,075-17,338) | $27,112  (17,553-36,672) | $17,339  (14,765-19,914) |
| **2018-2019** | $10,889  (10,066-11,711) | $7,178  (6,525-7,830) | $18,377  (16,344-20,409) | $29,510  (21,463-37,556) | $21,564  (17,059-26,068) |

Abbreviations: ASCVD: Atherosclerotic cardiovascular disease; HF: Heart failure

^a^ Analyses were not adjusted for covariates. Survey weights and procedures were utilized. See text for details on ascertainment of spending and definitions of cardiorenal conditions.

**Table A4:** Unadjusted trends in the mean (95% confidence interval) medication spending in persons with diabetes and cardiorenal comorbidities.^a^

| **Year** | **Diabetes overall** | **Diabetes w/o cardiorenal** | **Diabetes w /ASCVD** | **Diabetes w/ HF** | **Diabetes w/ kidney disease** |
| --- | --- | --- | --- | --- | --- |
| **2008-2009** | $3,737  (3,548-3,927) | $2,879  (2,668-3,089) | $5,184  (4,721-5,646) | $6,995  (5,741-8,249) | $6,592  (5,703-7,482) |
| **2010-2011** | $3,624  (3,417-3,831) | $2,905  (2,639-3,171) | $4,972  (4,583-5,362) | $6,118  (4,999-7,236) | $5,588  (4,953-6,223) |
| **2012-2013** | $3,880  (3,618-4,142) | $3,052  (2,761-3,344) | $5,298  (4,702-5,894) | $8,303  (6,601-10,005) | $6,690  (5,545-7,834) |
| **2014-2015** | $5,008  (4,236-5,781) | $4,225  (3,250-5,199) | $6,536  (5,293-7,778) | $7,502  (5,307-9,697) | $9,402  (4,686-14,118) |
| **2016-2017** | $5,260  (4,855-5,666) | $4,252  (3,838-4,667) | $7,306  (6,429-8,182) | $7,771  (5,934-9,608) | $8,677  (7,145-10,208) |
| **2018-2019** | $5,326  (5,021-5,632) | $4,184  (3,901-4,466) | $7,501  (6,678-8,325) | $10,205  (7,169-13,242) | $9,708  (8,625-10,792) |

Abbreviations: ASCVD: Atherosclerotic cardiovascular disease; HF: Heart failure

^a^ Analyses were not adjusted for covariates. Survey weights and procedures were utilized. See text for details on ascertainment of spending and definitions of cardiorenal conditions.
